# Mobile Phone Apps in the Management and Assessment of Mild Cognitive Impairment and/or Mild-to-Moderate Dementia: An Opinion Article on Recent Findings

**DOI:** 10.3389/fnhum.2017.00461

**Published:** 2017-09-15

**Authors:** Blanka Klimova

**Affiliations:** Department of Applied Linguistics, Faculty of Informatics and Management, University of Hradec Kralove Hradec Kralove, Czechia

**Keywords:** mobile phone apps, dementia, patients, benefits, limitations

## Introduction

Due to demographic changes, there has been a growing number of aging population. For example, in Europe older adults, aged 55+ years, form 25% of the whole population (Global AgeWatch Index 2015: Insight report., 2015). This increasing trend in the rise of older population groups brings about serious economic and social changes accompanied with a number of aging diseases such as dementia (Klimova et al., 2015).

At the moment, the prevalence of dementia reaches 58 million people worldwide, however, by 2050 it should triple since each year there are about 9.9 million of new dementia cases (Langa, 2015; WHO dementia, 2016). The key symptom of dementia is the global deterioration of cognitive functions, which is usually progressive and irreversible in nature. Other symptoms of dementia include a considerable loss of memory, orientation problems, impaired communication skills, depression, behavioral changes, and confusion (Craik and Salthouse, 2002; Salthouse, 2012). In addition, people with dementia suffer from behavioral disorders such as apathy, depression, delusions, hallucinations, aggression, irrelevant sexual behavior, or sleeping problems. All these symptoms impose a significant emotional and physical burden not only on the patients themselves, but also on their caregivers who have to look after these patients because they are dependent on their help (Hahn and Andel, 2011). In fact, 80% of the caregivers are family members whose quality of life rapidly declines after taking care of their loved ones suffering from dementia (Alzheimer's Association, 2015).

Nevertheless, in the early phases of dementia patients are still able to take care of themselves by modifying their life styles with little or no additional ongoing support, in which especially the availability and adoption of health-focused mobile phone apps can help both the patients and their caregivers with the self-management of dementia (Archer et al., 2014). Boulos et al. (2014) estimate that there are over 40,000 health-related apps on the market. However, Singh et al. (2016) argue that many mobile apps are not designed to meet the specific needs of patients with dementia and/or their caregivers. This finding has been also confirmed by Conci et al. (2009) who point out that older people are willing to accept and adopt a new technology if it meets their needs and expectations. The main barriers seem to be poor technical knowledge and skills, negative attitudes toward the use of this technology, as well as inaccurate perceptions of people with dementia (Klimova et al., 2016; O'Connor et al., 2016). Furthermore, there is a general lack of clinical trial evidence supporting the use of mobile apps (Singh et al., 2016), as well as interventions focusing on the needs of the dementia cohort of patient (Zhang et al., 2016).

Nevertheless, as Coppola et al. (2013) indicate, through proper training and appropriate strategies, technology can improve the quality of life of people with cognitive disorders. They also claim that mobile apps have the potential to reduce depression and stimulate cognitive functions. The very positive feature of mobile phones is that they do not have any stigma for the individuals who use or wear them (Joe and Demiris, 2013). Thanks to their ubiquitous nature, mobile phones can collect a huge number of various data (e.g., physiological or behavioral) in real time with limited burden on the client. These data can then provide decisions about treatment options and monitoring response (Arean et al., 2016).

Users of mobile phone apps can easily download the software programs to their mobile device with the Internet capability. These programs can be then used in several ways, which can help people with MCI and/or dementia to reduce their cognitive impairments (cf. Vardeh et al., 2013). The programs for the improvement of cognitive disorders in dementia are especially used for tracking wandering people via global posting system (GPS) (Faucounau et al., 2009), scheduling activities of their daily life or appointments at their doctor, or communication (Thorpe et al., 2016). Most of these mobile phone apps work on Android platforms, which allow users to change the sensitivity of the touch interface and can provide a vibration feedback when the screen is touched. Screen protectors can also help decrease glare (Coppola et al., 2013).

The purpose of this article is to explore the role of mobile phone apps for patients with mild cognitive impairment (MCI) and/or mild-to-moderate dementia and/or their caregivers. In addition, the author of this article highlights benefits and limitations of mobile phone apps for people suffering from cognitive disorders.

## Recent findings on the use of mobile apps for dementia

The findings from the recent research studies indicate that mobile phone apps can help patients with dementia in the enhancement of their quality of life by targeting these apps at their cognitive deficiencies such as spatial disorientation or memory loss (Lanza et al., 2014; Leng et al., 2014). In addition, as evidence shows, mobile phone apps can especially contribute to an early diagnosis and assessment of dementia (Onoda and Yamaguchi, 2014; Sangha et al., 2015; Zorluoglu et al., 2015). They can also assist caregivers who are in most cases patients' spouses in lowering their mental and economic burden (Thorpe et al., 2016) and learning more about their patients (Pitts et al., 2015). Generally, in the early phases of dementia, respectively mild-to-moderate stage of dementia, mobile phone apps can make these people independent and socially engaged.

Thorpe et al. (2016) explored the adoption of smartphone and smartwatch with six mobile apps (scheduling, navigation, communication, orientation, emergency help, and monitoring) among people with mild dementia. In their study they discovered that the smartphone should be used for input and smartwatch for output, e.g., notifications, orientation, and behavior sensing. The most appreciated function by patients was scheduling which reminded or notified them about the activities they should perform. The navigation or emergency support functions were not considered to be that much useful. The most important aspect for patients with dementia was personalization of both devices, i.e., tailoring the devices to their individual needs. This was in fact confirmed in other research studies as well (Faucounau et al., 2009; Lanza et al., 2014; Leng et al., 2014; Malinowsky et al., 2014). Faucounau et al. (2009) argue that it is a must to involve end-users in the co-design of new technologies in order to develop tailored devices, as well as testing them in a real-world context (Thorpe et al., 2016). Lanza et al. (2014) also point out that familiarization is one of the key aspects in the usability of these devices and apps by patients with dementia since patients with dementia are generally older people and they like to use what they already know. Yamagata and Kowto (2013) or Onoda et al. (2013) emphasize that the touch accuracy for these people might cause a difficulty and therefore technical aspects of the devices should be considered, e.g., weight of device, screen size, or the layout of buttons and taskbar. Thus, if all these features are taken into account, then the person-centered care can be provided as it has been discussed in Leng et al. (2014). Boulos et al. (2014) list the key ingredients necessary for a successful app. These include content quality, usability, need to match the app to consumer's general and health literacy levels, device connectivity standards, security and user privacy.

Furthermore, the findings of the research studies (Onoda and Yamaguchi, 2014; Sangha et al., 2015; Zorluoglu et al., 2015) show evidence that mobile phone apps are effective in cognitive screening and the assessment and diagnosis of dementia. The potential of mobile apps for the diagnosis and assessment of dementia is that these apps are more accurate than the traditional, manual testing; they are easily administered and understood by older people; some can be self-administered (cf. Onoda and Yamaguchi, 2014); they save time; they can minimize the examiner's biases; early diagnosis enables patients to stay independent on their tasks of daily living; they may cut potential costs on treatment and hospitalization; they target to improve the overall quality of life of older individuals; and they are ecological (cf. Klimova et al., 2017). For example, Onoda and Yamaguchi (2014) reported that their CADi2 had high sensitivity (0.85–0.96) and specificity (0.81–0.93) and were comparable with traditional neuropsychological tests such as MMSE or VTF. The same is true for Zorluoglu et al. (2015) study in which the findings of MCS and MoCA tests were compared, and the scores of individuals from these tests were correlated (*r*^2^ = 0.57). These results are also in compliance with Allard et al. (2014) who maintain that traditional devices are often not capable to detect sensitive declines in cognitive functions due to natural variation at the time of testing. In addition, mobile devices can provide repeated and reliable assessments of cognitive functions.

Table 1 below then summarizes the principal benefits and limitations of the use of mobile phone apps in the management and assessment of MCI and/or mild-to-moderate dementia.

**Table 1:** Benefits and limitations of the use of mobile phone apps in the management and assessment of MCI and/or mild-to-moderate dementia.

**Benefits**	**Limitations**
Potential cost savings of care, reduced mental burden for patient's caregiver; Activities of daily life become easier, which can lead to an improved quality of life for users and a reduced need for help from society and relatives; Patients and caregivers are positive and motivated to use technologies in the future; Provide both patients and their caregivers with safety and security; Offer opportunities for early assessment and diagnosis of dementia.	Still not sufficient access to these devices; More evidence on the efficacy of technologies is required; Unexpected ethical as well as environmental and health consequences of new technologies used in medicine; Improved knowledge and awareness of the benefits of these devices are needed.

*Source: author's own processing*.

## Conclusion

The author of this opinion article indicates that the mobile phone apps can provide support for patients with MCI and/or mild-to-moderate dementia in their activities of daily life, and especially, in the early diagnosis of this cognitive disorder. In addition, they can reduce both mental and economic burden of patients and their caregivers. Nevertheless, more evidence-based research studies should be conducted to prove the efficacy of mobile phone apps intervention in the management of cognitive disorders such as dementia to properly address this issue (cf. Boulos et al., 2014; Garcia-Casal et al., 2016) since more research is performed with healthy older individuals (cf. Kueider et al., 2012; Powers et al., 2013; Lampit et al., 2014; Toril et al., 2014).

And although older people will be becoming digitally more savvy, training should be provided to avoid social anxiety of using these mobile phone apps. In addition, companies developing health-related mobile phone apps should focus on meeting specific needs of their potential older clients whose number will be rapidly rising in the near future (cf. Thorpe et al., 2016).

## Author contributions

BK contributed to the drafting, analyses, and final version of the whole manuscript. The author read and approved the final manuscript.

### Conflict of interest statement

The author declares that the research was conducted in the absence of any commercial or financial relationships that could be construed as a potential conflict of interest. The reviewer KO and handling Editor declared their shared affiliation.

## References

[B1] Allard M. Husky M. Catheline G. Pelletier A. Dilharreguy B. Amieva H.. (2014). Mobile technologies in the early detection of cognitive decline. PLoS ONE 9:e112197. 10.1371/journal.pone.0112197 25536290 PMC4275158

[B2] Alzheimer's Association (2015). 2015 Alzheimer's disease facts and figures. Alzheimer's Dement. 11, 332–384. 10.1016/j.jalz.2015.02.003 25984581

[B3] Archer N. Keshavjee K. Demers C. Lee R. (2014). Online self-management interventions for chronically ill patients: cognitive impairment and technology issues. IJMI 83, 264–272. 10.1016/j.ijmedinf.2014.01.005 24507762

[B4] Arean P. A. Ly K. H. Andersson G. (2016). Mobile technology for mental assessment. Dialogues Clin. Neurosci. 18, 163–169. 27489456 10.31887/DCNS.2016.18.2/parean PMC4969703

[B5] Boulos M. N. K. Brewer A. C. Karimkhani C. Buller D. B. Dellavalle R. P. (2014). Mobile medical and health apps: state of the art, concerns, regulatory control and certification. OJPHI 5:e229. 10.5210/ojphi.v5i3.4814 24683442 PMC3959919

[B6] Conci M. Pianesi F. Zancanaro M. (2009). Useful, social and enjoyable: Mobile phone adoption by older people, in Proceedings of the 12th IFIP TC 13 International Conference on Human-computer Interaction: Part I. (Berlin; Heidelberg: Springer-Verlag), 63–76.

[B7] Coppola J. F. Kowtko M. A. Yamagata C. Joyce S. (2013). Applying mobile application development to help dementia and Alzheimer patients, in Wilson Center for Social Entrepreneurship. Paper 16. Available online at: http://digitalcommons.pace.edu/wilson (Accessed July 13, 2017).

[B8] Craik F. Salthouse T. (2002). The Handbook of Aging and Cognition, 2nd Edn. Mahwah, NJ: Lawrence Erlbaum.

[B9] Faucounau V. Riguet M. Orvoen G. Lacombe A. Rialle V. Extra J.. (2009). Electronic tracking system and wandering in Alzheimer's disease: a case study. Ann. Phys. Rehabil. Med. 52, 579–587. 10.1016/j.rehab.2009.07.034 19744906

[B10] Garcia-Casal J. A. Loizeau A. Csipke E. Franco-Martin M. Perea-Bartolome M. V. Orrell M. (2016). Computer-based cognitive interventions for people living with dementia: a systematic literature review and meta-analysis. Aging Ment. Health. 21, 454–467. 10.1080/13607863.2015.1132677 26806365

[B11] Global AgeWatch Index 2015: Insight report (2015). Available online at: http://www.population.gov.za/index.php/npu-articles/send/22-aging/535-global-agewatch-index-2015-insight-report (Accessed May 18, 2017).

[B12] Hahn E. A. Andel R. (2011). Non-pharmacological therapies for behavioral and cognitive symptoms of mild cognitive impairment. J. Aging Health 23, 1223–1245. 10.1177/0898264311422745 22086440

[B13] Joe J. Demiris G. (2013). Older adults and mobile phones for health: a review. J. Biomed. Inform. 46, 947–954. 10.1016/j.jbi.2013.06.008 23810858 PMC3836587

[B14] Klimova B. Maresova P. Valis M. Hort J. Kuca K. (2015). Alzheimer's disease and language impairments: Social intervention and medical treatment. Clin. Interv. Aging 10, 1401–1408. 10.2147/CIA.S89714 26346123 PMC4555976

[B15] Klimova B. Simonova I. Poulova P. Truhlarova Z. Kuca K. (2016). Older people and their attitude to the use of information and communication technologies – a review study with special focus on the Czech Republic (Older people and their attitude to ICT). Educ. Gerontol. 42, 361–369. 10.1080/03601277.2015.1122447

[B16] Klimova B. Valis M. Kuca K. (2017). Potential of mobile technologies and applications in the detection of mild cognitive impairment among older generation groups. Soc. Work Health Care 56, 588–599. 10.1080/00981389.2017.1316339 28463071

[B17] Kueider A. M. Parisi J. M. Gross A. L. Rebok G. W. (2012). Computerized cognitive training with older adults: a systematic review. PLoS ONE 7:e40588. 10.1371/journal.pone.0040588 22792378 PMC3394709

[B18] Lampit A. Hallock H. Valenzuela M. (2014). Computerized cognitive training in cognitively healthy older adults: a systematic review and meta-analysis of effect modifiers. PLoS Med. 11:e1001756. 10.1371/journal.pmed.1001756 25405755 PMC4236015

[B19] Langa K. M. (2015). Is the risk of Alzheimer's disease and dementia declining? Alzheimer's Res. Ther. 7, 1–4. 10.1186/s13195-015-0118-1 25815064 PMC4374373

[B20] Lanza C. Knorzer O. Weber M. Riepe M. W. (2014). Autonomous spatial orientation in patients with mild to moderate Alzheimer's disease by using mobile assistive devices: a pilot study. JAD 42, 879–884. 10.3233/JAD-140063 24958461

[B21] Leng F. Y. Yeo D. George S. Barr C. (2014). Comparison of iPad applications with traditional activities using person-centred care approach: impact on well-being for persons with dementia. Dementia 13, 265–273. 10.1177/1471301213494514 24339097

[B22] Malinowsky C. Nygard L. Kottorp A. (2014). Using a screening tool to evaluate potential use of e-health services for older people with and without cognitive impairment. Aging Ment. Health 18, 340–345. 10.1080/13607863.2013.832731 24548108

[B23] O'Connor S. Bouamrane M. M. O'Donnell C. A. Mair F. S. (2016). Barriers to co-desingning mobile technology with persons with dementia and their carers. Stud. Health Technol. Inform. 225, 1028–1029. 10.3233/978-1-61499-658-3-1028 27332466

[B24] Onoda K. Yamaguchi S. (2014). Revision of the Cognitive Assessment for Dementia, iPad version (CADi2). PLoS ONE 9:e109931. 10.1371/journal.pone.0109931 25310860 PMC4195614

[B25] Onoda K. Hamano T. Nabika Y. Aoyama A. Takayoshi H. Nakagawa T.. (2013). Validation of a new mass screening tool for cognitive impairment: cognitive Assessment for Dementia, iPad version. Clin. Interv. Aging 8, 353–360. 10.2147/CIA.S42342 23569368 PMC3615850

[B26] Pitts K. Pudney K. Zachos K. Maxden N. Krogstie B. Jones S. (2015). Using mobile devices and apps to support reflective learning about older people with dementia. Behav. Inform. Technol. 34, 613–631. 10.1080/0144929X.2015.1015165

[B27] Powers K. L. Brooks P. J. Aldrich N. J. Palladino M. Alfieri L. (2013). Effects of video-game play on information processing: a meta-analytic investigation. Psychol. Bull. Rev. 20, 1055–1079. 10.3758/s13423-013-0418-z 23519430

[B28] Salthouse T. (2012). Consequences of age-related cognitive declines. Annu. Rev. Psychol. 63, 201–226. 10.1146/annurev-psych-120710-100328 21740223 PMC3632788

[B29] Sangha S. George J. Winthrop C. Panchal S. (2015). Confusion: delirium and dementia - a smartphone app to improve cognitive assessment. BMJ Qual. Improv. Rep. 4:u202580.w1592. 10.1136/bmjquality.u202580.w1592 26732085 PMC4645948

[B30] Singh K. Drouin K. Newmark L. P. Filkus M. Silvers E. Bain P. A.. (2016). Patient-facing mobile apps to treat high-need, high-cost populations: a scoping review. JMIR Mhealth Uhealth 4:e136. 10.2196/mhealth.6445 27993761 PMC5206484

[B31] Thorpe J. R. Ronn-Andersen K. V. Bien P. Ozkil A. G. Forchhammer B. H. Maier A. M. (2016). Pervasive assistive technology for people with dementia: a UCD case. Health Technol. Lett. 3, 297–302. 10.1049/htl.2016.0057 28008366 PMC5168821

[B32] Toril P. Reales J. M. Ballesteros S. (2014). Video game training enhances cognition of older adults? A meta-analytic study. Psychol. Aging 29, 706–716. 10.1037/a0037507 25244488

[B33] Vardeh D. Edwards R. R. Jamison R. N. Eccleston C. (2013). There's an app for that: Mobile technology is a new advantage in managing chronic pain. Pain 21, 1–7.

[B34] WHO dementia (2016). Available online at: http://www.who.int/features/factfiles/dementia/en/ (Accessed May 18, 2017).

[B35] Yamagata C. Kowto M. (2013). Mobile app development and usability research to help dementia and Alzheimer patients, in Systems, Applications and Technology Conference (LISAT). (Long Island, NY).

[B36] Zhang M. W. B. Chan S. Wynne O. Jeong S. Hunter S. Wilson A.. (2016). Conceptualization of an evidence-based smartphone innovation for caregivers and persons living with dementia. Technol. Health Care 24, 769–773. 10.3233/THC-161165 27129032

[B37] Zorluoglu G. Kamasak M. E. Tavacioglu L. Ozanar P. O. (2015). A mobile application for cognitive screening of dementia. Comput. Methods Programs Biomed. 118, 252–262. 10.1016/j.cmpb.2014.11.004 25481217

